# Association between hair loss severity and risk for later mental health problems in women irradiated for tinea capitis in childhood

**DOI:** 10.1192/j.eurpsy.2021.475

**Published:** 2021-08-13

**Authors:** D. Segal-Engelchin, S. Shvarts

**Affiliations:** 1 Social Work, Ben-Gurion University of the Negev, Beer-Sheva, Israel; 2 Faculty Of Health Sciences, Ben-Gurion University of the Negev, Beer Sheva, Israel

**Keywords:** hair loss severity, mental health problems, irradiation treatment, women

## Abstract

**Introduction:**

Hair loss resulting from childhood irradiation for tinea capitis has been linked to mental health effects in women. However, the association of hair loss severity with mental health in this population is unknown.

**Objectives:**

The aim of this study is to examine the association between hair loss severity and mental health outcomes in women irradiated for tinea capitis in childhood and to identify contributing factors to these outcomes.

**Methods:**

Medical records, held at the archives of Israel National Center for Compensation of Scalp Ringworm Victims, were retrospectively reviewed for 2509 women who received compensation for full or partial alopecia resulting from irradiation in childhood for tinea capitis. Mental health outcomes were determined by the number of mental health conditions reported.

**Results:**

Among women with high hair loss levels, risk was increased for a range of mental health problems, including depression symptoms, emotional distress, social anxiety, low self-esteem, and suicidal ideation. Hair loss severity emerged as a significant predictor of mental health, adding to the effects of other predictors such as family, and social and physical health problems. Effects of hair loss severity on mental health outcomes were mediated by women’s negative social experiences.

**Conclusions:**

Hair loss severity is a significant risk factor for mental health problems in women irradiated for tinea capitis in childhood. Further research is needed to assess mental health risks among women with severe hair loss associated with additional diseases.

**Disclosure:**

No significant relationships.

